# Long-term efficacy and safety of adjunctive perampanel in pediatric patients aged 4–19 years with epilepsy: a real-world study

**DOI:** 10.1038/s41598-023-40594-8

**Published:** 2023-09-01

**Authors:** Song Ee Youn, Hoon-Chul Kang, Joon Soo Lee, Heung Dong Kim, Se Hee Kim

**Affiliations:** 1 Department of Pediatrics, Yonsei University College of Medicine, Division of Pediatric Neurology, Severance Children’s Hospital, 50-1 Yonsei-ro, Seodaemun-gu, Seoul, 03722 Republic of Korea; 2 Department of Pediatrics, Kangdong Sacred Heart Hospital, Hallym University College of Medicine, 150 Seongan-ro, Gangdong-gu, Seoul, 05355 Republic of Korea

**Keywords:** Neuroscience, Neurology

## Abstract

This study determined the 24-month outcomes of perampanel treatment in children and adolescents with epilepsy. The percentage of ≥ 50% responders was 47.3% (139/294) at 12 months and 49.0% (144/294) at 24 months. A 100% reduction in seizures for more than 12 months was observed in 12.2% (36/294). Discontinuation occurred in 39.8% (117/294). The most common reason for discontinuation was adverse events (29.1%, 34/117). Baseline seizure frequency was higher in children aged < 12 years than in patients aged ≥ 12 years; however, the percentage of seizure reduction and ≥ 50% responders did not significantly differ between the two groups. The rate of early discontinuation was higher (*p* < 0.001) and the duration of perampanel treatment was shorter in children aged < 12 years (*p* = 0.001). Most children aged < 12 years discontinued PER due to inadequate effectiveness, while adverse event was the most common reason in patients aged ≥ 12 years (*p* = 0.045). Only slow titration was significantly associated with ≥ 50% of responders. In conclusion, this study showed that perampanel can be utilized effectively and safely for a prolonged period in pediatric patients aged 4 to < 12 years, as well as in patients aged 12 years and older.

## Introduction

Perampanel (PER) is the first highly-selective, non-competitive antagonist of the alpha-amino-3-hydroxy-5-methyl-4 isoxazole propionic acid (AMPA) receptor, which is approved as an anti-seizure medication. PER inhibits the generation, maintenance, and spread of epileptiform activity by blocking glutamate AMPA receptors at postsynaptic excitatory synapses^1,2^.

Since PER was first approved to treat partial (focal) onset seizures in adult patients in 2012, its indication has been extended. Now, it is approved to treat primary generalized tonic–clonic seizures in people with epilepsy who are 12 years of age and older. It has also been approved to treat partial (focal) onset seizures in people with epilepsy who are 4 years of age and older.^3^.

Few studies evaluated the efficacy and safety of PER in children. In these studies, about 40% and 65% of the patients had 50% or higher seizure reduction based on their epilepsy types^4–6^. However, these 3-month and 1-year studies evaluated short-term efficacy. These could have overestimated the anti-seizure efficacy of PER, especially in patients with scarce seizures. Data regarding the long-term use of PER for children remain to be investigated.

Here, we determined the 24-month outcomes of PER treatment in children and adolescents with epilepsy. We also compared the efficacy and tolerability between pediatric and adolescent patients.

## Methods

### Patients

This study was a retrospective, observational, single-center study. Patients with epilepsy who received PER as an adjunctive treatment at Severance Children’s Hospital from February 2016 to February 2021 were included. The inclusion criteria of patients were as follows: (1) aged ≥ 4 years and < 19 years at the time of PER initiation, (2) ≥ 24 months of follow-up period, (3) had at least one seizure within 3 months before the first PER intake. We excluded patients whose accurate seizure history or frequency data were not available.

### Efficacy and safety analyses

For efficacy, ≥ 50%, ≥ 90%, and 100% responder rates were analyzed. Seizure frequency was compared to that during the baseline period. “Baseline” was defined as 3 months before PER treatment. Patients regularly visited the clinic every 1 to 3 months. Therefore, monthly mean seizure frequency was collected at 3-month intervals from the initiation of PER treatment at 3, 6, 9, 12, 15, 18, 21, and 24 months.

Good responders to PER treatment were considered as patients with ≥ 50% reduction in seizure frequency. No responders included patients with a reduction of < 50% in seizure frequency. Patients who had epilepsy surgeries or changes of baseline anti-seizure medications during the 24 months were also counted as no responders. Various clinical factors, including age at the initiation of PER, sex, PER dose, titration speed, number of concomitant anti-seizure drugs, epilepsy type, etiology, seizure onset age, seizure type, baseline seizure frequency, and delayed development, were evaluated to find an association with good seizure outcome. Patients were considered to undergo slow dose titration if PER was increased by 2 mg at intervals of more than 2 weeks or longer.

Adverse events were graded according to the Common Terminology Criteria for Adverse Events (CTCAE) version 5.0 (U.S. Department of Health and Human Services, 2017). Patients who discontinued PER during the 24-month period were counted for tolerability, and reasons for discontinuation were collected.

### Statistical analyses

Seizure outcomes were analyzed using both observed and imputed data. For imputed data, the last observation carried forward (LOCF) analysis and intention to treat (ITT) analysis were performed. Wilcoxon rank-sum test was used to compare continuous variables, and chi-squared test or Fisher’s exact test was used to compare categorical variables. Our modeling logit link was used to deal with longitudinal binary outcome measurements. Logistic regression was performed to identify the factors associated with ≥ 50% seizure reduction at 24 months. Furthermore, univariate and multivariate analyses were used to find an association with good seizure outcomes. Kaplan Meier plot and log-rank test were performed to evaluate the risk of discontinuation between age subgroups. Data are expressed as a median (interquartile range) or number (%), as appropriate. A *p-*value < 0.05 was considered statistically significant. All statistical analyses were performed using the SAS software, version 9.4 (SAS Institute Inc., Cary, NC, USA.).

### Ethical approval

This study was supported by the Severance Hospital Yonsei University and approved by the Institutional Review Board of Severance Children’s Hospital (IRB No. 4-2016-0684). The need for informed consent was waived by the Severance Hospital Yonsei University and approved by the Institutional Review Board of Severance Children’s Hospital due to the study being retrospective in nature.

All methods were carried out in accordance with relevant guidelines and regulations.

## Results

### Patient characteristics

This study consisted of 294 patients. Table 1 presents the patient demographics and characteristics at baseline. The median age at PER initiation was 14.2 (12.1–16.2) years. Patients aged < 12 years were 65 (22.1%). The most common patient group was developmental and epileptic encephalopathy including Lennox-Gastaut syndrome, comprising 110 patients (37.4%), followed by 110 patients (37.4%) with focal epilepsy and nine patients (3.1%) with generalized epilepsy. The most common cause of epilepsy was malformation of cortical development (20.7%), followed by hypoxic-ischemic encephalopathy (18.0%), genetic (11.9%), and encephalitis (7.5%). The median baseline monthly seizure frequency was 20.0 (3.6–90.0).

**Table 1:** Patient characteristics (*n* = 294).

Characteristics	Values (%)
Male	166 (56.5)
Follow-up period (month)	50.7 (39.8–58.9)
Age at seizure onset (month)	29.0 (6.0–77.1)
Age of perampanel initiation (year)	14.2 (12.1–16.2)
Duration of perampanel treatment (month)	35.3 (5.9–52.9)
Number of concomitant ASMs	3.0 (2.0;4.0)
Baseline seizure frequency (per month)	20.0 (3.6;90.0)
Etiology	
Malformation of cortical development	61 (20.7)
Hypoxic-ischemic encephalopathy	53 (18.0)
Genetic	35 (11.9)
Encephalitis	22 (7.5)
Metabolic	12 (4.1)
Tuberous sclerosis complex	8 (2.7)
Tumor	4 (1.4)
Unknown	99 (33.7)
Diagnosis	
DEE	148 (50.3)
Focal epilepsy	110 (37.4)
Generalized epilepsy	9 (3.1)
Unclassified	27 (9.1)
Epilepsy surgery	132 (44.9)
Resective epilepsy surgery	69 (23.5)
Corpus callosotomy	37 (12.6)
Vagus nerve stimulation	26 (8.8)
Maximum dose of perampanel (mg/day)	6.0 (4.0;8.0)

Data are expressed as number (percent) or median (interquartile range).

ASMs, anti-seizure medications; DEE (Developmental and Epileptic Encephalopathy).

The median follow-up duration after PER initiation was 50.7 (39.8–58.9) months, and the median treatment duration with PER was 35.3 (5.9–52.9) months.

### Efficacy outcomes related to PER therapy

**Figure 1:**
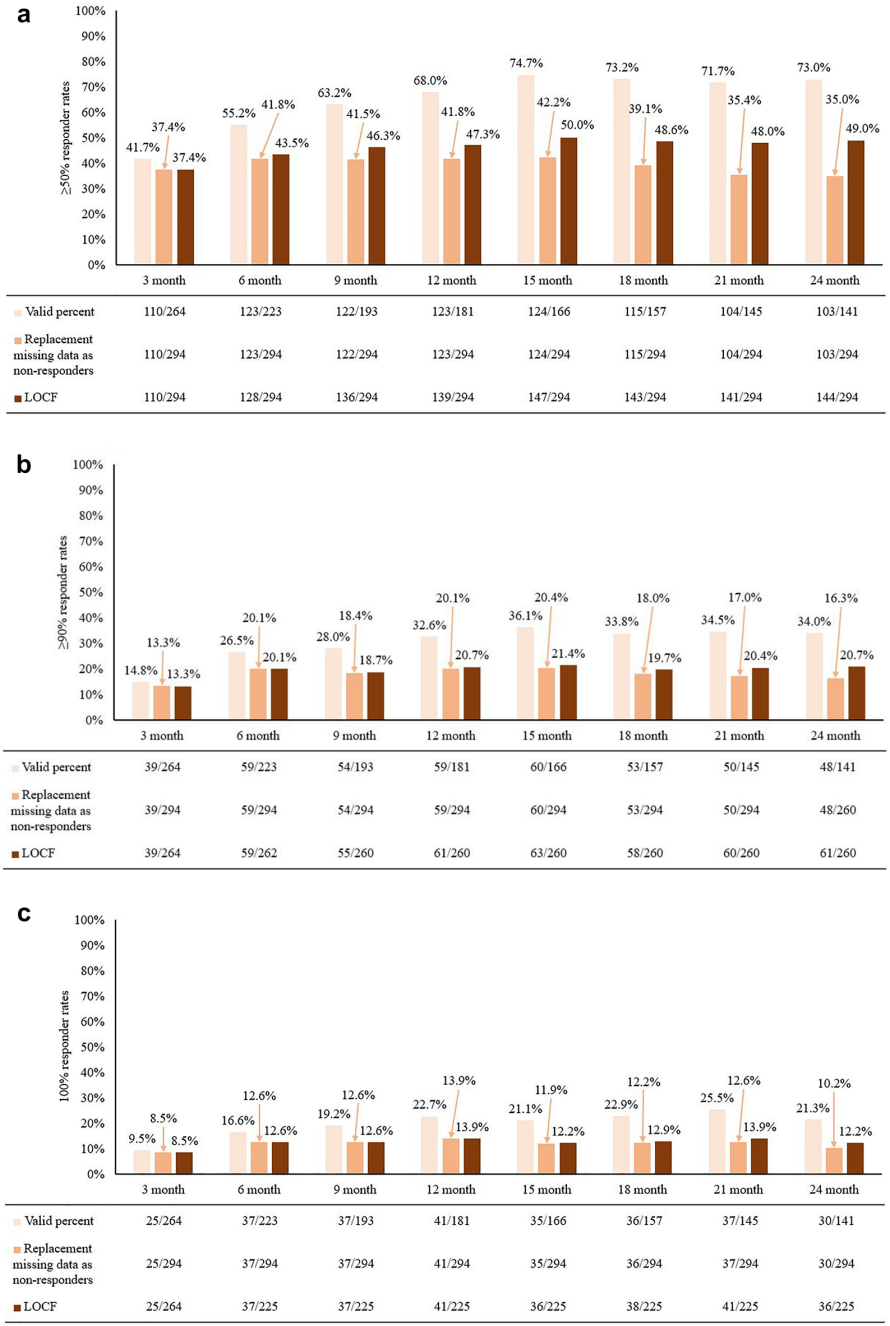
Outcomes related to perampanel therapy. (**a**) ≥ 50% responder rates during the 24-month treatment period. (**b**) ≥ 90% responder rates during the 24-month treatment period. (**c**) 100% responder rates during the 24-month treatment period. LOCF, last observation carried forward.

The percentage of ≥ 50% responders increased from 41.7% at baseline to 73% at 24 months.The percentage of ≥ 90% responders was 34.0% at 24 months. A 100% reduction in seizures was observed in 36 patients (12.2%) at 24 months (Fig. 1). Among 132 patients who had epileptic surgery before PER initiation, nine patients achieved a 100% reduction in seizures with PER.

When the LOCF imputation method was used, the percentage of ≥ 50% responders was 47.3% at 12 months and 49.0% at 24 months. The ≥ 90% responder rates slightly reduced from 20.1% at 6 months to 16.3% at 24 months (Fig. 1).

Overall, the median percentage of seizure frequency reduced by 40.9% at 24 months from the baseline when the LOCF imputation method was used. Seizure frequency reduced significantly at 24 months compared to the baseline (*p* < 0.001).

Early discontinuation occurred in 117 patients (39.8%). The most common reason for discontinuation was adverse events (34/117, 29.1%), followed by an inadequate therapeutic response (31/117, 26.5%) and seizure aggravation (29/117, 24.8%).

### Comparison between age groups

**Figure 2:**
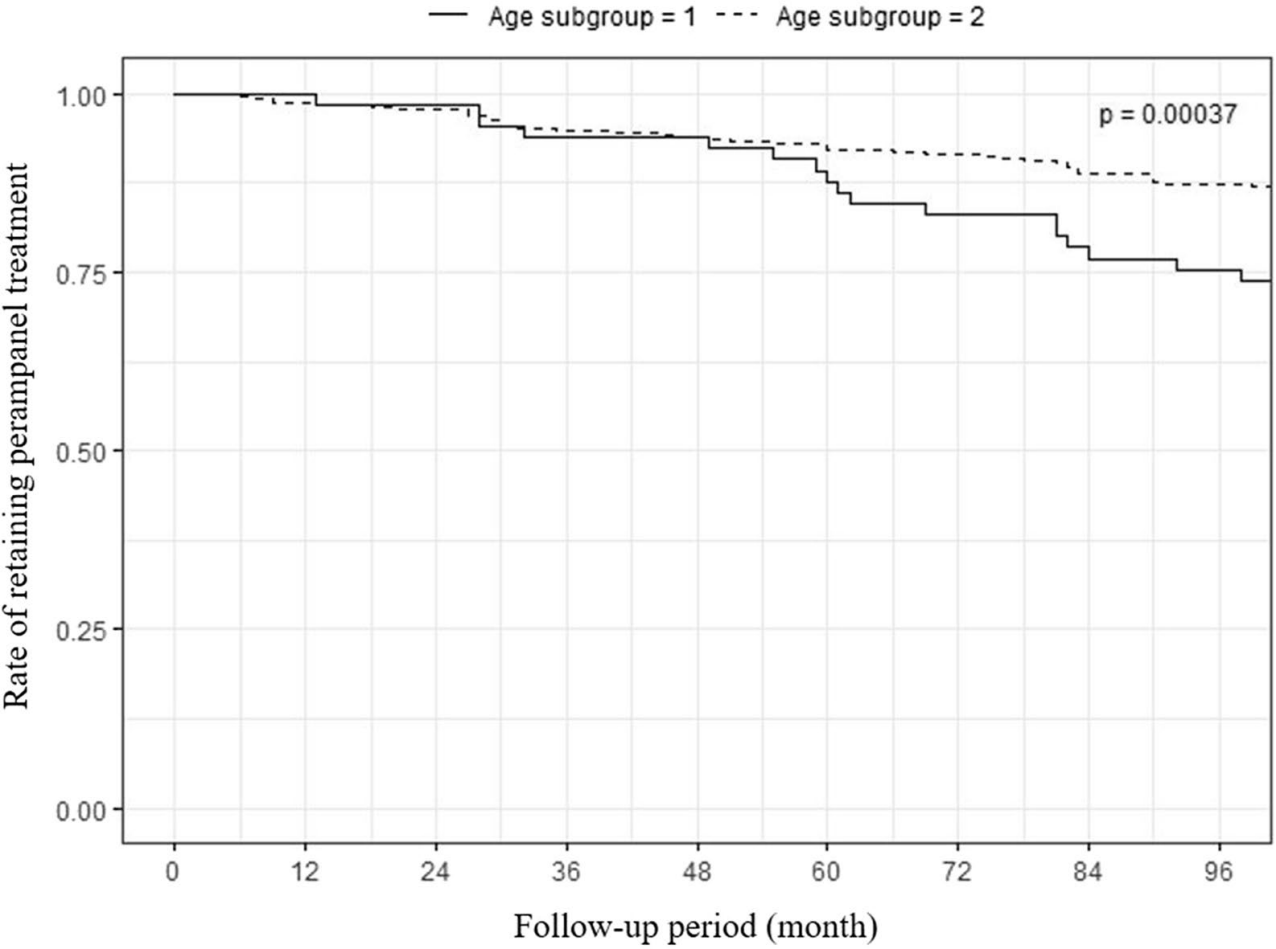
Kaplan–Meier curve for time to early treatment discontinuation. The rate of early discontinuation was higher (*p* < 0.001) and the duration of perampanel treatment was shorter in children aged <12 years (*p* = 0.001). Solid line indicates children aged <12 years (age subgroup 1) and dotted line indicates patients aged ≥12 years (age subgroup 2).

We compared children aged < 12 years with patients aged ≥ 12 years (Table 2). The percentage of seizure reduction and ≥ 50% responder rates did not significantly differ between the two groups during the follow-up period. However, the rate of early discontinuation was higher (*p* < 0.001), and the duration of PER treatment was shorter in children aged < 12 years (*p* = 0.001) (Fig. 2).

**Table 2:** Difference between children and adolescents.

Variables	4 years ≤ age < 12 years (n = 65)	≥ 12 years (n = 229)	*p*-value
Male			
Follow-up period (month)	44.4 [30.6;61.5]	51.4 [41.3;58.6]	0.138
Age at seizure onset (month)	15.0 [5.5;61.0]	36.0 [6.0;84.5]	0.029
Duration of perampanel treatment (month)	24.0 [3.1;36.2]	40.3 [10.0;53.9]	< 0.001
Number of concomitant ASMs	3.0 [2.5;4.0]	3.0 [2.0;4.0]	0.074
Baseline seizure frequency (per month)	60.0 [10.0;157.5]	12.0 [3.0;75.0]	< 0.001
Diagnosis			0.172
DEE	39 (60.0)	109 (47.6)	
Focal epilepsy	17 (26.2)	93 (40.6)	
Generalized epilepsy	1 (1.5)	8 (3.5)	
Unclassified	8 (12.4)	19 (8.3)	
Adverse events	19 (29.2)	107 (46.7)	0.012
Adjustment for adverse events			0.049
Withdrawal of perampanel	9 (47.4)	25 (23.4)	
Reduction of perampanel dose	7 (36.8)	31 (29.0)	
No management	3 (15.8)	51 (47.7)	
Early discontinuation of perampanel	36 (55.4)	81 (35.4)	0.004
Reason for discontinuation of perampanel			0.045
Ineffectiveness	15 (45.5)	16 (26.2)	
Seizure aggravation	9 (27.3)	20 (32.8)	
Adverse effect	9 (27.3)	25 (41.0)	
Not described	3 (8.3)	20 (24.7)	

Data are expressed as number (percent) or median (interquartile range).

ASMs, anti-seizure medications; DEE (Developmental and Epileptic Encephalopathy).

The most common reason for discontinuation was also different. Most children aged < 12 years discontinued PER due to inadequate effectiveness, while adverse event was the most common reason for PER discontinuation in patients aged ≥ 12 years (*p* = 0.045) (Table 2).

### Adverse events and safety

**Figure 3:**
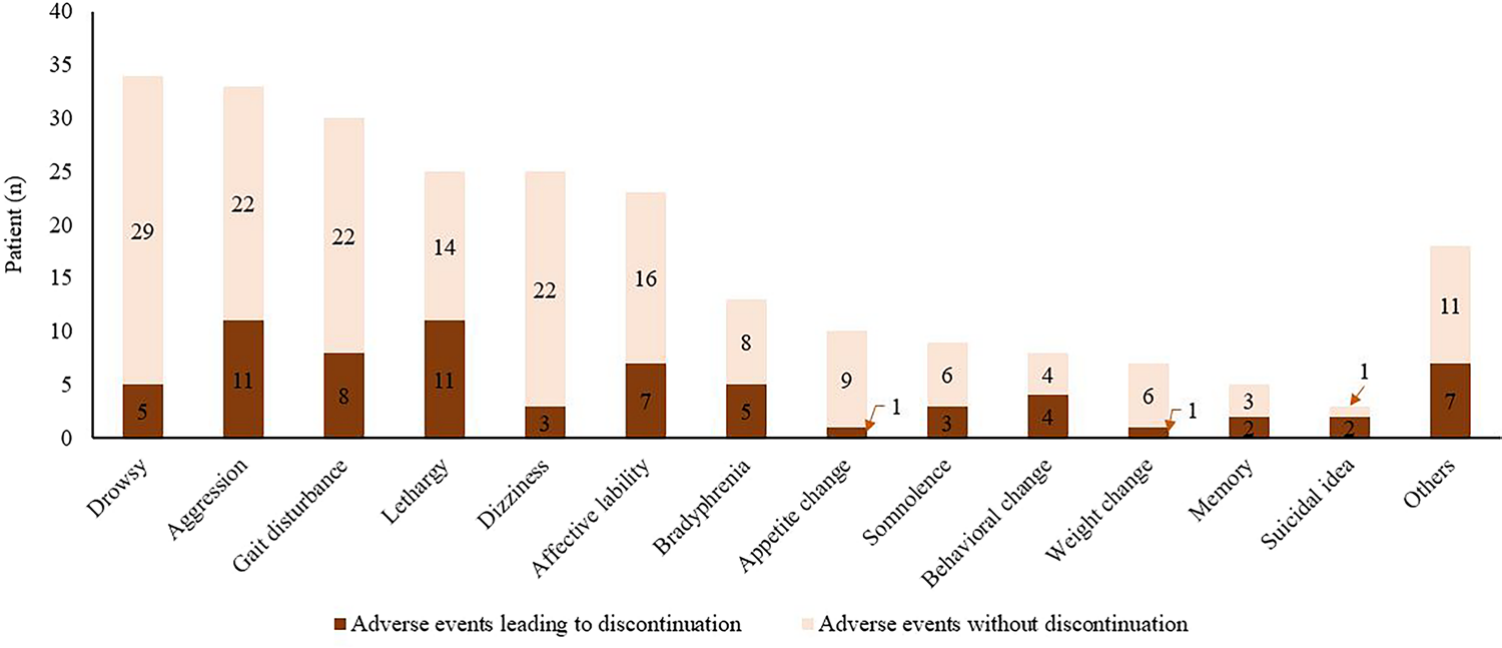
Adverse events.

One hundred twenty-six patients (42.9%) experienced adverse events. The most common adverse event was drowsiness (34 patients, 11.6%), followed by aggressive behavior (33 patients, 11.3%) and gait disturbance (30 patients, 10.2%). The most common adverse events leading to discontinuation were aggressive behavior and lethargy (Fig. 3). Other cases with adverse events were temporary or had minimal effects on daily activities. Most of these adverse events were resolved spontaneously or after reducing the dose or slowing titration.

### *Factors associated with* ≥ 50% reduction in seizures

We investigated the factors associated with ≥ 50% seizure reduction at 24 months. Higher maximum and maintenance dose, slow titration, and etiology were factors associated with ≥ 50% of responders in univariate analysis. However, in multivariate analysis, only slow titration was significantly associated with ≥ 50% seizure reduction. Patients with good responders used the PER treatment longer (*p* < 0.001). (Table 3).

**Table 3:** Univariate and multivariate analyses of factors associated with ≥ 50% seizure reduction at 24 months.

Variables	Univariate		Multivriate	
	OR (95% CI)	*P*	OR (95% CI)	*P*
Male	0.817 (0.543,1.229)	0.331		
Age of seizure onset (month)	0.998 (0.994,1.002)	0.416		
Adolescent	1.347 (0.786,2.310)	0.278		
Delayed development	0.968 (0.747,1.255)	0.806		
Epilepsy syndrome	1.057 (0.885,1.263)	0.540		
Etiology	1.185 (1.002,1.401)	0.048	1.151 (0.952,1.392)	0.146
Number of concomitant ASMs	0.841 (0.699,1.011)	0.066		
Baseline seizure frequency (per month)	1.001 (1.000,1.002)	0.206		
Seizure type	1.005 (0.748,1.351)	0.973		
Age of perampanel initiation (year)	0.996 (0.952,1.041)	0.847		
Duration of treatment (month)	1.043 (1.032,1.054)	< 0.001	1.044 (1.031,1.057)	< 0.001
Slow titration	2.016 (1.324,3.070)	0.001	1.739 (1.047,2.889)	0.033
Dose (mg/day)				
Initial	1.009 (0.777,1.311)	0.945		
Maximun	1.096 (1.026,1.170)	0.006	0.943 (0.797,1.118)	0.501
Maintenance	1.111 (1.035,1.191)	0.003	1.057 (0.894,1.250)	0.517

OR; odds ratio, CI; confidence interval, ASMs; antiseizure medications.

## Discussion

The anti-seizure effect of PER was sustained during 24-month treatment in children and adolescents with drug-resistant epilepsy. Seizure reduction rates of 100% and 50% responder rates were comparable to those observed in previous short-term studies^4,5^. Remarkably, fewer adverse events and higher percentage of discontinuation due to ineffectiveness were observed in pediatric patients compared to adolescents, showing that the optimal pattern of PER use may be different among these patients.

In our ITT analysis, the 50% responder rate was 35% after 24 months of PER treatment, and a 100% reduction in seizure was achieved in 10%. In the LOCF analysis, the 50% responder and 100% reduction rates were higher and reached 49% and 12%, respectively. Observed data analysis showed that the highest 50% responder and 100% reduction rates at 24 months were 73% and 21%, respectively. These numbers were comparable to previous studies on pediatric patients; in a study which included pediatric patients aged 4 to < 12 years, 50% responder rates were 47% and 65% for focal seizures and focal bilateral tonic–clonic seizures, respectively^4^. Another study reported 50% responder rates of 44% after 12-month treatment of PER in pediatric patients aged 4 years or older^5^. The 100% reduction rates ranged between 18 and 36% in these previous studies. The reported rates were higher than those in our data, as these studies were not performed in an ITT manner^4–7^; however, similar sustainability was observed in our study. A long-term study that included adolescents reported that the overall anti-seizure effect of PER was maintained for 48 months^8,9^.

When children were compared with adolescents, the outcomes of seizure reduction were not different between the two groups in our study. Previous studies have reported similar responses to PER between adults and adolescents^8,10^. Also, high responder rates similar to those of adults have been reported in children^4,7^. No significant difference in efficacy has been observed in patients of different ages^5,7^. Our study extends these findings, confirming that the efficacy of PER is sustained for 24 months regardless of the patient’s age group.

In our study, adverse events occurred in 43% of the patients. Previous short-term studies reported similar rates ranging between 16 and 40% in children and adolescents^5,7,11^. Common treatment-emergent adverse events (TEAEs) were drowsiness, aggressive behavior, and gait disturbance, and no unusual adverse events were observed in the study, different from adult studies. Previous studies reported higher TEAEs ranging between 74 and 100%, probably due to the study’s prospective nature^4,6,12,13^; however, most of the TEAEs were temporary and resolved after reducing doses or titration speed^5,6^. The overall incidence of adverse events leading to discontinuation was low^4^. These findings show that PER can be used safely in children and adolescents over a long-term period.

Notably, the group aged aged 4 to < 12 years had fewer adverse events compared to the group aged ≥ 12 years. This finding did not correlate well with the previous ones. One study reported that TEAEs occurred more frequently in patients aged 4 to < 7 years than in patients aged 7 to < 12 years^4^. Another study reported a similar incidence of TEAEs between patients aged 2 to < 7 years and 7 to < 12 years^6^. This discrepancy may have occurred due to the different patient populations and the different sizes of the studies. Our study included patients with severe drug-resistant epilepsy. Indeed, in the group aged 4 to < 12 years, 60% of the patients had Lennox-Gastaut syndrome. These patients with developmental epileptic encephalopathies may not have reported certain adverse events, such as dizziness or aggression. Generally, PER efficacy is not influenced by age, changes in body weight, or liver function^4^. The pharmacokinetic properties of PER in children aged ≤ 2 to < 12 years are independent of age and weight of age. More studies are warranted to confirm these findings.

The overall discontinuation rate was 40%. The most common reason for discontinuation was adverse events (36.2%), followed by inadequate effect (33.0%) and aggravation (30.9%).

The group aged 4 to < 12 years had higher discontinuation rates than the group aged ≥ 12 years, and ineffectiveness was the major reason for discontinuation in the group aged 4 to < 12 years. Again, this difference could have been attributed to the high percentage of patients with Lennox-Gastaut syndrome in the group aged 4 to < 12 years. It would be more challenging to treat these patients with severe drug-resistant epilepsy than to treat simple focal onset seizures in adolescents. In an open-label study, the most common primary reason for discontinuation was adverse events in children aged 2 to < 12 years, although adverse events led to discontinuation in a few patients (8%)^4^. We may have reduced such adverse events with slow titration. Slow titration of PER at a slow interval of 2 weeks or longer can reduce adverse events and discontinuation rate^14^.

This study had some limitations. There was selection bias. The study included patients with severe drug-resistant epilepsy and several comorbidities; this could have underestimated the anti-seizure effect of PER. Additionally, this was a retrospective study. However, it should also be highlighted that this was a real-world study performed in typical clinical situations.

In conclusion, this study shows that PER can be utilized effectively and safely for a prolonged period in pediatric patients aged 4 to < 12 years, as well as in patients aged 12 years and older.

## Data Availability

The dataset generated and analyzed in the current study is available from the corresponding author upon reasonable request.
